# Supplementary material for: Prevalence and predictors of COVID-19 vaccination hesitancy among healthcare workers in Sub-Saharan Africa: A systematic review and meta-analysis
Source: PLoS One. 2023 Jul 28;18(7):e0289295. doi: 10.1371/journal.pone.0289295 (PMC10381063; doi:10.1371/journal.pone.0289295)
Supplement: S1 Table — (DOCX) [file pone.0289295.s002.docx]

**Table S1: Search strategy for the databases**

**PubMed (Result-11206)**

| **SN** | **QUERRY** |
| --- | --- |
| #1 | ‘’Covid-19’’ OR ‘’SARS Cov-2’’ OR 2019-nCov |
| #2 | vaccination OR immunization |
| #3 | ‘’acceptance rate’’ OR hesitancy OR ‘’Hesitancy rate’ |
| #4 | ‘’healthcare workers’’ OR “health professional” OR HCW OR doctors OR Nurses OR Midwives OR Pharmacist OR Physiotherapist OR ‘’eye health workers’’ OR ‘’medical laboratory scientist’’ OR ‘’medical students’’ OR Dietician OR Dietitian OR “allied health professional” |
| #5 | Sub-Saharan Africa OR Africa |
| #6 | 1 AND 2 AND 3 AND 4 AND 5 |
| #7 | Limited to January 1, 2020 to October 31, 2022 |
| #8 | Limited to English |
| #9 | 6 AND 7 AND 8 |

**Search strategy for Google scholar (6410 results)**

| #1 | With all of the words | "Vaccination Hesitancy" OR "Vaccination reluctance" OR "Vaccination acceptance" |
| --- | --- | --- |
| #2 | With the exact phrase | “Healthcare workers” OR “Health professional” |
| #3 | With at least one of the words | “COVID 19" OR SARS Cov-2" OR "2019 nCov" OR Africa” |
| #4 | Anywhere in the article | #1 AND #2 AND #3 Limit to return articles dated between 2020 and 2022 |

**Search strategy for Science Direct (2622)**

| #1 | “Predictors” OR “Associated factors” OR “Determinants” |
| --- | --- |
| #2 | “COVID-19 vaccination hesitancy" OR "COVID-19 vaccination reluctance") OR "COVID-19 vaccination acceptance" |
| #3 | "Health workers" OR "Healthcare workers" |
| #4 | #1 AND #2 AND #3 AND #4 Limit by time span 2020-2022 |

**Search strategy for African Journals Online (Results=49)**

"Vaccination Hesitancy" OR "Vaccination acceptance" AND “Healthcare workers” OR “Health professional” AND “COVID 19" OR “SARS Cov-2" OR "2019 nCov" AND “Africa”
