# Supplementary material for: Prevalence and predictors of COVID-19 vaccination hesitancy among healthcare workers in Sub-Saharan Africa: A systematic review and meta-analysis
Source: PLoS One. 2023 Jul 28;18(7):e0289295. doi: 10.1371/journal.pone.0289295 (PMC10381063; doi:10.1371/journal.pone.0289295)
Supplement: S2 Table — (DOCX) [file pone.0289295.s003.docx]

**Table S2: Quality assessment checklist (adapted from Hoy et al [1])**

| Name of author(s):  Year of publication:  Study title: |  | |
| --- | --- | --- |
| **Risk of bias items** | **Risk of bias levels** | **Points scored** |
| 1. Was the study’s target population a close representation of the national population in relation to relevant variables, e.g. age, sex, occupation? | **Yes** (**LOW RISK**): The study’s target population was a close representation of the national population. | 0 |
|  | **No** (**HIGH RISK**): The study’s target population was clearly NOT representative of the national population. | 1 |
| 2. Was the sampling frame a true or close representation of the target population? | **Yes** (**LOW RISK**): The sampling frame was a true or close representation of the target population. | 0 |
|  | **No** (**HIGH RISK**): The sampling frame was NOT a true or close representation of the target population. | 1 |
| 3. Was some form of random selection used to select the sample, OR, was a census undertaken? | **Yes** (**LOW RISK**): A census was undertaken, OR, some form of random selection was used to select the sample (e.g. simple random sampling, stratified random sampling, cluster sampling, systematic sampling).  **No**    (  **HIGH RISK**  ):  A census was NOT undertaken, AND some form o  f  1 | 0 |
|  | random selection was NOT used to select the sample.  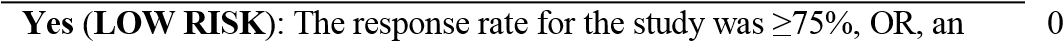  analysis was performed that showed no significant difference in relevant demographic characteristics between responders and non- responders  **No**    (  **HIGH RISK**  ):  The response rate was <75%, and if any  analysis  1    comparing responders and non-responders was done, it showed a significant difference in relevant demographic characteristics between responders and non-responders |  |
| 4. Was the likelihood of non-response bias minimal? |  |  |
| 5. Were data collected directly from the subjects (as opposed to a proxy)? | **Yes** (**LOW RISK**): All data were collected directly from the subjects. | 0 |
|  | **No** (**HIGH RISK**): In some instances, data were collected from a proxy. | 1 |
| 6. Was an acceptable case definition used in the study? | **Yes** (**LOW RISK**): An acceptable case definition was used.  **No** (**HIGH RISK**): An acceptable case definition was NOT used | 0  1 |
| 7. Was the study instrument that measured the parameter of interest (e.g. prevalence of low back pain) shown to have reliability and validity (if necessary)? | **Yes** (**LOW RISK**): The study instrument had been shown to have  reliability and validity (if this was necessary), e.g. test-re- test, piloting, validation in a previous study, etc. | 0 |
|  | **No** (**HIGH RISK**): The study instrument had NOT been shown to have reliability or validity (if this was necessary). | 1 |
| 8. Was the same mode of data collection used for all subjects? | **Yes** (**LOW RISK**): The same mode of data collection was used for all subjects. | 0 |
|  | **No** (**HIGH RISK**): The same mode of data collection was NOT used for all subjects. | 1 |
| 9. Were the numerator(s) and denominato r(s) for the parameter of interest appropriate | **Yes** (**LOW RISK**): The paper presented appropriate numerator(s) AND denominator(s) for the parameter of interest (e.g. the prevalence of low back pain). | 0 |
|  | **No** (**HIGH RISK**): The paper did present numerator(s) AND denominator(s) for the parameter of interest but one or more of these were inappropriate. | 1 |
| 10. Summary on the overall risk of study bias | **LOW RISK** | 0-3 |
|  | **MODERATE RISK** | 4-6 |
|  | **HIGH RISK** | 7-9 |

1. Hoy D, Brooks P, Woolf A, Blyth F, March L, Bain C, et al. Assessing risk of bias in prevalence studies: modification of an existing tool and evidence of interrater agreement. J Clin Epidemiol. 2012;65: 934-939.
