# Supplementary material for: Prevalence and predictors of COVID-19 vaccination hesitancy among healthcare workers in Sub-Saharan Africa: A systematic review and meta-analysis
Source: PLoS One. 2023 Jul 28;18(7):e0289295. doi: 10.1371/journal.pone.0289295 (PMC10381063; doi:10.1371/journal.pone.0289295)
Supplement: S1 Fig — (DOCX) [file pone.0289295.s005.docx]

**Figure S1. Leave one out sensitivty analysis**
