# Supplementary material for: Prevalence and predictors of COVID-19 vaccination hesitancy among healthcare workers in Sub-Saharan Africa: A systematic review and meta-analysis
Source: PLoS One. 2023 Jul 28;18(7):e0289295. doi: 10.1371/journal.pone.0289295 (PMC10381063; doi:10.1371/journal.pone.0289295)
Supplement: Attachment — Submitted filename: Prevalence and Predictors of COVID.docx [file pone.0289295.s006.docx]

Prevalence and Predictors of COVID-19 vaccination hesitance among healthcare workers in Sub-Saharan Africa: a systematic review and meta-analysis.

Minor comments

1. In abstract correct this sentence. Data were extracted using was extracted using excel.
2. Topographical errors like grammar and spelling correct it.

Major comments

1. Only cross sectional studies included what about case control, and cohort studies?
2. What about studies written other than English language?
3. All the studies regardless of the level of risk were included. So what is the significance of quality assessment, if you included all studies without assessing risk of bias. It is better to avoid those studies high risk of bias or weak studies and reanalyze?
