# Supplementary material for: Prevalence and predictors of COVID-19 vaccination hesitancy among healthcare workers in Sub-Saharan Africa: A systematic review and meta-analysis
Source: PLoS One. 2023 Jul 28;18(7):e0289295. doi: 10.1371/journal.pone.0289295 (PMC10381063; doi:10.1371/journal.pone.0289295)
Supplement: Attachment — Submitted filename: PLOS ONE SYTEMATIC REVIEW.docx [file pone.0289295.s007.docx]

**PLOS ONE REVIEW**

Prevalence and Predictors of COVID-19 vaccination hesitance among healthcare workers in Sub-Saharan Africa: a systematic review and meta-analysis

**--Manuscript Draft--**

**Manuscript Number: PONE-D-22-29316**

Article Type: Research Article

**Full Title:** Prevalence and Predictors of COVID-19 vaccination hesitance among healthcare workers in Sub-Saharan Africa: a systematic review and meta-analysis

**Short Title:** Prevalence and Predictors of COVID-19 vaccination hesitance

**Abstract**

The abstract speaks to the systematic review. The problem is stated with relevant support the systematic review. The methods shows summary of the articles used but were limited to cross-sectional studies of a period of 2 years (2020-2022). This is reasonable period since the COVID-19 pandemic started in 2019 achieving appropriate results. The abstract concludes with summary of the findings relevant to the subject. The results are pertinent to the local region

**Background**

1. The review was not of randomized trials. The review included the description of the approaches used to identify all the literature in the subject. The question the paper wants to answer should be stated clearly. Moreover, it is surprising that the review could not capture the study below which was conducted in a West African country among a specific healthcare professionals about COVID -19 hesitancy in vaccine uptake to give more credence to the systematic study. It is advised the authors include this paper in the review unless they found it in the eleven articles removed as stated in the results.

***Botwe BO, Antwi WK, Adusei JA, Mayeden RN, Akudjedu TN, Sule SD. COVID-19 vaccine hesitancy concerns: Findings from a Ghana clinical radiography workforce survey. Radiography (Lond). 2022 May;28(2):537-544. Doi: 10.1016/j.radi.2021.09.015. Epub 2021 Oct 8. PMID: 34654631; PMCID: PMC8498685*.**

2. The context of the systematic review including why it’s important are stated.
3. The statement in page (8-9) “COVID-19 vaccine hesitance and acceptance among Healthcare workers in Sub-Saharan Africa have been investigated through observational research [4]” cannot be totally true as the study quoted above was not an observational study.

**Why Methods and methods?**

Looking at the period of the unset of the pandemic the participants identified in the 22 articles and the aggregate data used is large enough for the systematic review.

**Search Engine**

Appropriate but would be good to include SCOPUS articles if possible.

**Eligibility criteria**

**Inclusion**

The review included only studies reporting on predictors of COVID-19 vaccination hesitance. However, Botwe et al study indicated above reported similar findings

**Results**

Data was synthesized.

1. The writers offer a high-quality, well-protected setting for assessing significant research works that advance understanding of the subject matter and a specific geographic area.
2. The study’s characteristics are displayed clearly in the results and supported with Forest plot of the pooled proportion of COVID-19 vaccine hesitance.
3. However, the Table with the heading ***“Predictors of COVID-19 vaccine hesitance among Health workers in Sub-Saharan Africa”*** is supposed to come with a label (**i.e Table 2)**
4. Overall the results will help local health policies

**Discussion.**

The discussion was tailored to the results and in relation to previous studies used for the systematic review.
