# Supplementary material for: Prevalence and predictors of COVID-19 vaccination hesitancy among healthcare workers in Sub-Saharan Africa: A systematic review and meta-analysis
Source: PLoS One. 2023 Jul 28;18(7):e0289295. doi: 10.1371/journal.pone.0289295 (PMC10381063; doi:10.1371/journal.pone.0289295)
Supplement: Attachment — Submitted filename: Response to reviewers.docx [file pone.0289295.s008.docx]

Eustes Kigongo

Department of Environmental Health and Disease Control, Lira University

Uganda

June 15, 2023

Dear Editor,

**Re: Response to reviewers’ comments**

First all, I wish to thank the reviewers and editors for the positive feedback that will help to improve the quality of our manuscript.

This letter serves to re-submit our manuscript titled “**Prevalence and Predictors of COVID-19 vaccination hesitance among healthcare workers in Sub-Saharan Africa: a systematic review and meta-analysis**”**.**

Please see the responses in the table below;

| **Sn** | **Comments** | **Response** | **Page, Lines** |
| --- | --- | --- | --- |
| 1 | Please amend your authorship list in your manuscript file to include all the authors. | Thank you for the good observation,  The authorship list has been added. | Pg 1,  Lines 3-4 |
| 2 | Please amend your list of authors on the manuscript to ensure that each author is linked to an affiliation. | Thank you for the good observation,  The authors affiliations have been added in the manuscript. | Pg 1,  Lines 6-15 |
| 3 | We note that some of your files are duplicated on your submission. Please remove any unnecessary or old files from your revision, and make sure that only those relevant to the current version of the manuscript are included. | Thank you,  The files have been updated. | NA |
