# Supplementary material for: Prevalence and predictors of COVID-19 vaccination hesitancy among healthcare workers in Sub-Saharan Africa: A systematic review and meta-analysis
Source: PLoS One. 2023 Jul 28;18(7):e0289295. doi: 10.1371/journal.pone.0289295 (PMC10381063; doi:10.1371/journal.pone.0289295)
Supplement: Attachment — Submitted filename: Response to Reviewers.docx [file pone.0289295.s010.docx]

Eustes Kigongo

Department of Environmental Health and Disease Control, Lira University

Uganda

July 14, 2023

Dear Editor,

**Re: Response to reviewers’ comments**

First all, I wish to thank the reviewers and editors for the positive feedback that will help to improve the quality of our manuscript.

This letter serves to re-submit our manuscript titled “**Prevalence and Predictors of COVID-19 vaccination hesitance among healthcare workers in Sub-Saharan Africa: a systematic review and meta-analysis**”**.**

Please see the responses in the table below;

| SN | Reviewers’ comments | Response | Page/lines |
| --- | --- | --- | --- |
| 1 | Abstract: Please ensure that the results are not repeated in the conclusion. The conclusion should provide a concise summary of the findings and highlight their implications and significance | Thank you,  We have removed the results from the conclusion. | Pg 1,  Lines 35-36 |
| 2 | Please review the order of keywords to ensure consistency and alignment with relevant concepts in the manuscript. | Thank you,  The key words have been reorganized as advised. | Pg 1,  Lines 40 |
| 3 | Ensure consistent use of terms related to vaccine hesitancy throughout the manuscript to maintain clarity and coherence. | For consistency, we have used “Hesitancy” throughout the entire manuscript. | All pages |
| 4 | Please provide a comprehensive and well-defined set of exclusion criteria that clearly outline the criteria for excluding studies from the systematic review. | Thank you,  We have provided the exclusion criteria. | Pg 4,  Lines 123-125 |
| 5 | Please clarify if the tool adapted from Hoy et al. has been validated. If any item was removed, provide a justification for its exclusion. In addition, please include the questions and scoring criteria for the risk of bias assessment in the supplementary file, as requested previously. | This has been clarified, we used the validated tool and we did not modify.  The tool has been attached as supplementary file 3. | Pg 5,  Lines 139-140 |
| 6 | Please use the correct symbol (p<0.05) to indicate statistical significance, rather than using "P=0.05." | Thank you,  This has been rectified. | Pg 5,  Lines 158 |
| 7 | Include the search strategies for Google Scholar, African Journal Online, and Science Direct in the supplementary file, along with the date and results in numbers. Currently, the supplementary file only includes the search strategy for PubMed. | Thank you,  This has been addressed accordingly. Attached as Supplementary file 1 | NA |
| 8 | Please provide full names for the abbreviations used in the Supplementary File 2 and ensure that the citations contain the corresponding references. | Thank you,  This has been done. | NA |
| 9 | Please revise the first sentence of the conclusion to accurately reflect the quantitative data and align it with the findings of the study. | Thank you,  This has been revised. | Pg 14,  Lines 311-312 |
| 10 | Please carefully review the comments provided by Reviewer 4 in the attached PDF and address them accordingly. | Thank you for the comments,  Comments in the attached document (manuscript) have been reviewed. | All document |
